# Protein Stability Perturbation Contributes to the Loss of Function in Haploinsufficient Genes

**DOI:** 10.3389/fmolb.2021.620793

**Published:** 2021-02-01

**Authors:** Giovanni Birolo, Silvia Benevenuta, Piero Fariselli, Emidio Capriotti, Elisa Giorgio, Tiziana Sanavia

**Affiliations:** ^1^ Department of Medical Sciences, University of Torino, Italy; ^2^ Department of Pharmacy and Biotechnology (FaBiT), University of Bologna, Italy; ^3^ Department of Molecular Medicine, University of Pavia, Italy

**Keywords:** protein mutation, protein stability, haploinsuffciency, variant effect prediction, protein stability prediction

## Abstract

Missense variants are among the most studied genome modifications as disease biomarkers. It has been shown that the “perturbation” of the protein stability upon a missense variant (in terms of absolute ΔΔG value, i.e., |ΔΔG|) has a significant, but not predictive, correlation with the pathogenicity of that variant. However, here we show that this correlation becomes significantly amplified in haploinsufficient genes. Moreover, the enrichment of pathogenic variants increases at the increasing protein stability perturbation value. These findings suggest that protein stability perturbation might be considered as a potential cofactor in diseases associated with haploinsufficient genes reporting missense variants.

## Introduction

Missense variations may cause loss-of-function by directly perturbing protein-protein interactions or ablating enzymatic activity or by inducing structural destabilization of the protein (Stein et al., 2019), which in turn may trigger protein misfolding and degradation. Many neurodegenerative diseases, such as Parkinson’s disease, are also associated with destabilization of the corresponding proteins (Wilson et al., 2014). However, there are cases where missense variations increase protein stability while still being deleterious. As an example, the variation H101Q in the CLIC2 protein has been associated with a mental disorder and predicted to make the CLIC2 protein thermodynamically more stable and to interact more strongly with the ryanodine receptor, obstructing its transport to the cell membrane (Witham et al., 2011). Therefore, stability perturbations, rather than protein destabilization, can be linked with disease-causing variations.

Recently, Gerasimavicius et al. have highlighted an improvement in the identification of pathogenic variations using |ΔΔG| values (Gerasimavicius et al., 2020). However, very little is known about thermodynamic changes in human protein variants so far (Sanavia et al., 2020), and the processes establishing whether a variation perturbing the protein stability is or not disease-related are not clear yet. An extensive comparative analysis has proven that, on average, variations mostly involved in disease also associated with large effects on protein stability (Casadio et al., 2011). Although several studies tried to predict the functional or structural impacts of missense variations, the mechanism of the phenotypic impact through inheritance modes of the missense variations are still unclear. Indeed recessive variations are mainly observed in the buried region of protein structures and more likely associated with loss-of-function, whereas dominant variations are significantly enriched in the interfaces of molecular interactions and more difficult to be identified as disease-related (Guo et al., 2013; Martelli et al., 2016).

One of the most known pathogenic mechanisms for loss-of-function mutations is haploinsufficiency, a type of genetic dominance wherein a single functional copy of a gene is insufficient to maintain normal function. Different theories have been put forth to explain the cause of haploinsufficiency. One of them states that growth defects caused by changes in gene dosage are due to stoichiometric imbalances of protein complexes interfering with cellular functions (Veitia and Potier, 2015), whose interactions relying on the relative stoichiometry may be either cooperative or competitive. An example of this latter case is the cytotoxic T‐lymphocyte‐associated protein 4 (CTLA4), which competes for the same ligands with cluster of differentiation 28 (CD28), a T‐cell activator. An inappropriate balance of CTLA4 and CD28 can result in T‐cell overactivation by CD28 and autoimmune disease. Recently, it was observed a fatal heterozygous mutation in CTLA‐4, predicted to decrease protein stability resulting in haploinsufficiency and decreased CTLA‐4 expression in a patient reporting autoimmunity (Evan’s syndrome), lymphoproliferation and severe infections (Moraes-Fontes et al., 2017).

In this brief report, we suggest that one possible contribution to the pathogenic mechanism in haploinsufficient genes can be related to missense variants perturbing protein stability.

## Method

### Dataset

Performance assessment of 13 computational stability predictors, i.e., FoldX 5.0 (Delgado et al., 2019), INPS3D (Savojardo et al., 2016), Rosetta (Alford et al., 2017), PoPMusic (Dehouck et al., 2011), I-Mutant (Capriotti et al., 2005), SDM (Worth et al., 2011), SDM2 (Pandurangan et al., 2017), mCSM (Pires et al., 2014a), DUET (Pires et al., 2014b), CUPSAT (Parthiban et al., 2006), MAESTRO (Laimer et al., 2016), ENCoM (Frappier et al., 2015), DynaMut (Rodrigues et al., 2018), was investigated for detecting pathogenicity in (Gerasimavicius et al., 2020), considering |ΔΔG| values obtained from each predictor on a dataset of 13,508 missense variations from 96 different high-resolution (<2 Å) crystal structures of disease-associated monomeric proteins encoded by 100 genes. The dataset includes 3,338 missense variants which are annotated in Clinvar (Landrum et al., 2018) as pathogenic or likely pathogenic, associated to proteins with at least 10 known pathogenic missense variations occurring at residues present in the structure. These pathogenic variants are compared against 10,170 “putatively benign” missense variants collected from gnomAD v2.1 (Karczewski et al., 2020) from the same genes as the pathogenic variants. In order to highlight whether the performance obtained by the protein stability predictors might be influenced by the inheritance mode of the related coding genes, we annotated them according to the curated lists of autosomal dominant/recessive and haploinsufficient genes reported by the MacArthur Lab (https://github.com/macarthur-lab/gene_lists). The number of variants for each inheritance mode, split by pathogenic/benign, are 1,217/1,252, 753/1,819, and 635/4,253 for haploinsufficient, dominant, and recessive genes, respectively.

### Performance Evaluation

The assumption is that the |ΔΔG| values provided by the predictors can be used as a measure of pathogenicity, with lower values associated with neutral variations. The |ΔΔG| values are used to compute the area under the receiver operating characteristic curve (AUC) as the performance metric as in (Gerasimavicius et al., 2020). In this way, we do not need to select any specific threshold for the perturbation to define a pathogenicity score. However, to avoid biases due to the low proportion of pathogenic variants, here the AUC and the precision were calculated by averaging the results on balanced subsets. More precisely, the available pathogenic variants were matched with a random subset with the same number of benign variants for 100 times. This procedure was applied to the full dataset, for each gene separately and for the variants of each specific inheritance mode (i.e. haploinsufficient, autosomal dominant and recessive), along with their complement set. AUCs were always computed on |ΔΔG| values.

## Results

**FIGURE 1:**
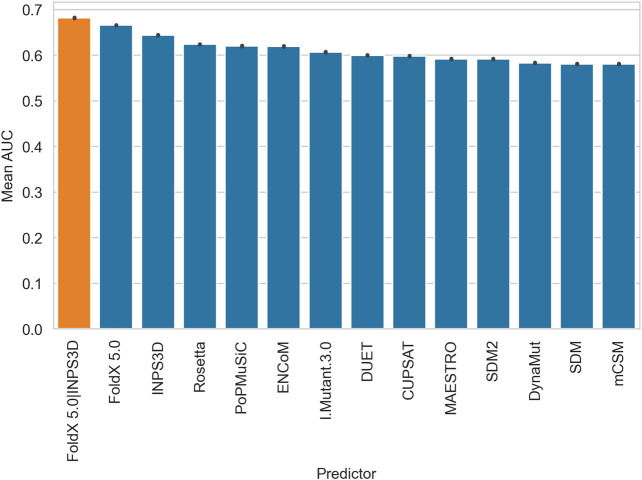
Barplots displaying the performance (AUC) of all the ΔΔG predictors and the consensus (orange) of the best two performing methods (FoldX5.0 and INPS3D). The bars represent the mean AUC obtained by averaging balanced subsets (the available pathogenic variants were matched with a random sample with the same number of benign variants for one hundred times).

Figure 1 shows the AUCs obtained from each predictor and the mean output of the best two performing methods (FoldX 5.0 and INPS3D, orange bar in the figure). We also tested all combinations of the three best predictors, which performed slightly worse (Supplementary Figure S1,S2). The bars reported in Figure 1 reflect the probability of a randomly chosen disease variant being assigned a higher-ranking score than a random benign one (Gerasimavicius et al., 2020). The barplots highlight the variability in terms of performance among the prediction stability-based methods, with FoldX 5.0 reaching the best AUC. It is worth noting that the combination of the scores from FoldX 5.0 and INPS3D increases the AUC performance of 2 percentage points over FoldX 5.0.

**FIGURE 2:**
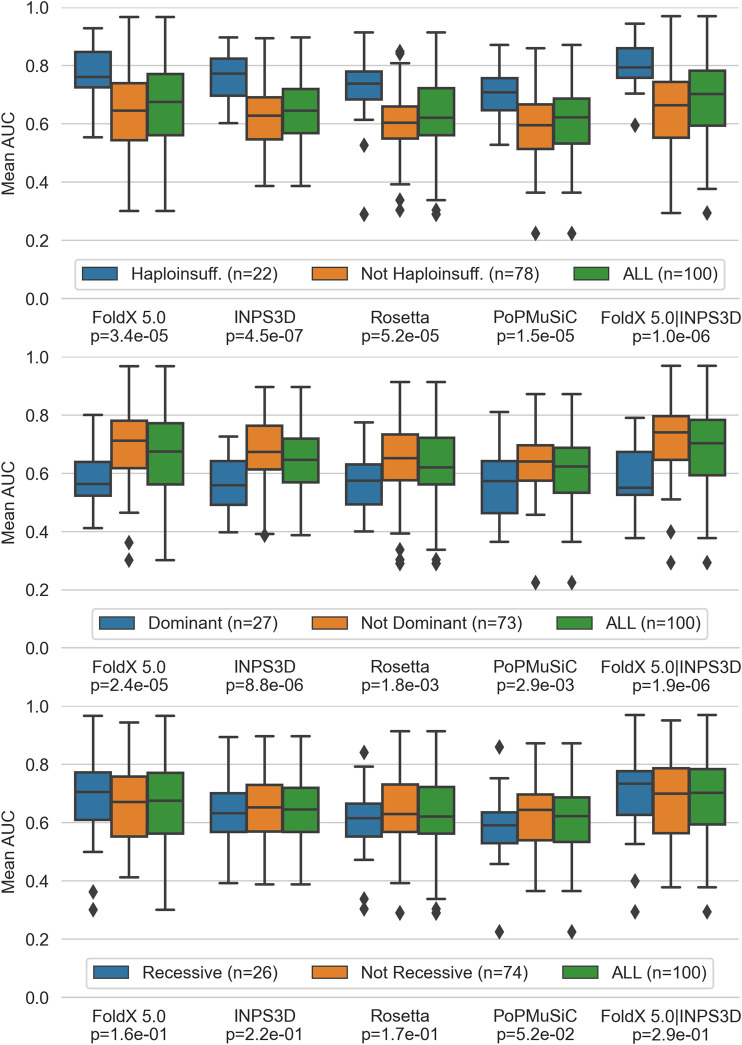
Performance of top performing predictors, (i.e. FoldX 5.0 and INPS3D, Rosetta and PoPMuSiC along with the combined scores of the first two) split by haploinsufficient, dominant without haploinsufficiency and recessive genes. P-values of the pairwise comparison between each gene group and its complement by the Mann-Whitney *U* test are reported at the bottom of the *x*-axis.

We then evaluated the scores by grouping the gene variants according to their inheritance mode (i.e. autosomal dominant/recessive or haploinsufficiency) in order to provide a biological interpretation. Interestingly, we found that the performance is significantly higher in haploinsufficient genes (Figure 2, top panel), while it is lower in not haploinsufficient dominant genes (Figure 2, central panel). Recessive genes show no significant differences from non-recessive genes. (Figure 2, central and bottom panels).

**FIGURE 3:**
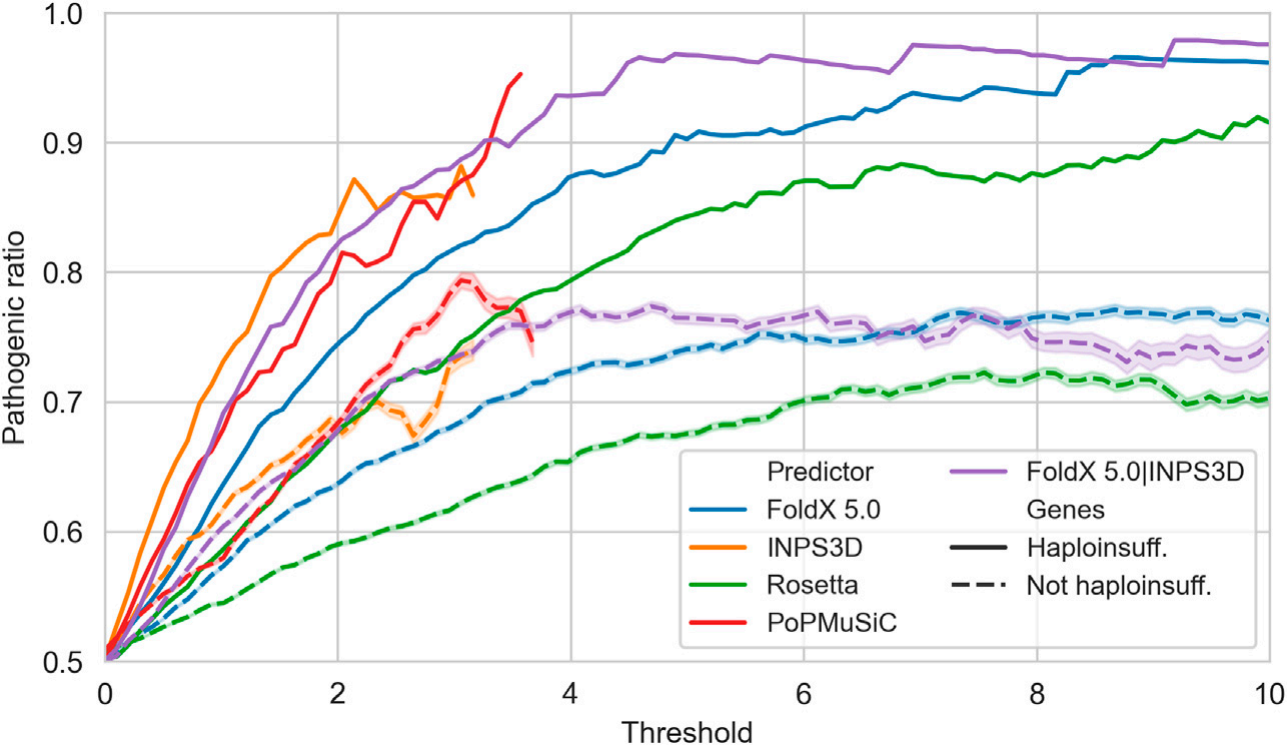
Precision (*y*-axis) of the protein stability-based methods in predicting pathogenicity at different |ΔΔG| values, defined as the ratio of truly pathogenic over all the variants reporting predicted |ΔΔG| values above a specific threshold (*x*-axis). Solid and dashed lines are computed on variants in haploinsufficient and non-haploinsufficient genes, respectively. INPS3D and PoPMuSiC lines stop earlier since the methods do not provide predictions with |ΔΔG| values greater than the reported thresholds.

Since stability change is one of the possible disease mechanisms to be linked with potential pathogenicity, we do not expect a high predictive power for small ΔΔG values. However, we can expect an enrichment of pathogenic variants at increasing protein stability perturbations. This hypothesis is confirmed in Figure 3, where we observed that variants with very high |ΔΔG| values tend to be strongly enriched in pathogenic variants. In general this is valid for all genes, but much more for haploinsufficient genes.

This result suggests that it is possible to generate a highly specific test for pathogenicity by selecting the variants according to a fixed threshold for the predicted |ΔΔG|. However, choosing the best |ΔΔG| threshold is highly dependent on the type of predictor used. When considering the best performing one, i.e., the mean between FoldX 5.0 and INPS3D |ΔΔG| values, we see that a threshold of 4.4 kcal/mol yields a precision (positive predictive value) of 96%.

Most of the variants are predicted to be destabilizing by the predictors, and this prevents us from analyzing the effect of the stabilizing variants separetely. Conversely, when only the predicted destabilizing variants are considered (Supplementary Figure S3), the trends are similar but slightly higher to those reported in Figure 3.

## Discussion

Genetic dominance originates from a variety of unrelated mechanisms (Veitia and Potier 2015). One of those is haploinsufficiency, namely the intolerance of a gene to the loss of one allele. As a consequence, the relative protein dosage is half of the normal level, which is not sufficient to ensure a normal function and consequently causes the pathological phenotype. Possible genetic causes are, for example, the deletion of one allele or protein-truncating variants that may induce nonsense-mediated decay of transcripts.

The better performance of ΔΔG predictors in haploinsufficient genes suggests that missense variants causing significant changes in protein stability may play a relevant role in disease development when genes are haploinsufficient. It does not seem far-fetched to argue that variants causing strong ΔΔG perturbations are likely to yield a non-functional protein, thus becoming loss-of-function variants, which are the main driver of pathogenicity in haploinsufficient genes. On the other hand, the lower performance on non-haploinsufficient dominant genes shows that this role does not extend to other dominance mechanisms, which are often activated by “gain-of-function” variants, where the mutated protein actively interferes with the gene function. This may suggest that ΔΔG perturbations are not predictive of “gain-of-function” effects.

Figure 3 shows that protein stability-based methods are able to predict pathogenic variants in haploinsufficient genes at high precision (>96%) using thresholds on |ΔΔG| values above 4.4 kcal/mol. However, since ΔΔG perturbation is only one of the many molecular mechanisms affecting pathogenicity, we do not expect to gain in sensitivity by decreasing the |ΔΔG| threshold: missense variants predicted to cause only modest ΔΔG changes may cause disease by other mechanisms like compromising the protein interaction capabilities. On the other hand, significant ΔΔG perturbations can shift the protein far from its dynamically active state, making the protein non-functional. Indeed, we confirmed that perturbing variants (predicted to be either very destabilizing or stabilizing) have a high probability of being pathogenic. Thus, by choosing an appropriate |ΔΔG| threshold (which is dependent on the specific ΔΔG predictor), we can turn ΔΔG predictors into highly precise pathogenicity predictors for haploinsufficient genes.

While the absolute value of the ΔΔG was used for all analyses, it would have been interesting to analyze variants predicted to increase or decrease stability separately. This would have allowed us to check if stabilizing variants could be associated for instance with gain-of-function mechanisms, differently from destabilizing variants. However, a high proportion of the variants in our dataset were predicted to be destabilizing, leaving an insufficient number of stabilizing and especially highly stabilizing variants for a robust statistical analysis. This interesting question should be addressed in the next future when more data will be available by correctly mapping annotated variants to protein structures.

In conclusion, large ΔΔG perturbations in haploinsufficient gene products appear to be a significant factor in the pathogenicity assessment of the missense variants. Therefore, we recommend complementing the state-of-the-art pathogenicity predictions with one of the best performing ΔΔG predictors, at least for haploinsufficient genes, when looking for possible disease causes. High |ΔΔG| values indicate that protein stability perturbation is a reasonable cause of the observed pathological condition.

## Data Availability Statement

Publicly available datasets were analyzed in this study. These data can be found here: https://doi.org/10.1038/s41598-020-72404-w.

## Author Contributions

TS, PF and EC designed the research. GB retrieved the data and the annotations, ran the analyses and drafted the manuscript. All the authors interpreted the results and contributed to the submitted version.

## Funding

This work was supported by the PRIN project, “Integrative tools for defining the molecular basis of the diseases: Computational and Experimental methods for Protein Variant Interpretation” of the Ministero Istruzione, Università e Ricerca (201744NR8S). We thank the EU projects GenoMed4All (H2020 RIA n.101017549) and BRAINTEASER (H2020 RIA n.101017598).

## Conflict of Interest

The authors declare that the research was conducted in the absence of any commercial or financial relationships that could be construed as a potential conflict of interest.
