# Supplementary material for: Protein Stability Perturbation Contributes to the Loss of Function in Haploinsufficient Genes
Source: Front Mol Biosci. 2021 Feb 1;8:620793. doi: 10.3389/fmolb.2021.620793 (PMC7882701; doi:10.3389/fmolb.2021.620793)
Supplement: Supplementary file 1 [file datasheet1.pdf]

Supplementary materials for:

Protein stability changes upon missense variants may affect haplo-insufficient genes.

Giovanni Birolo(1), Silvia Benevenuta(1), Piero Fariselli(1)*, Emidio Capriotti(2)*, Elisa Giorgio(3), Tiziana Sanavia(1)

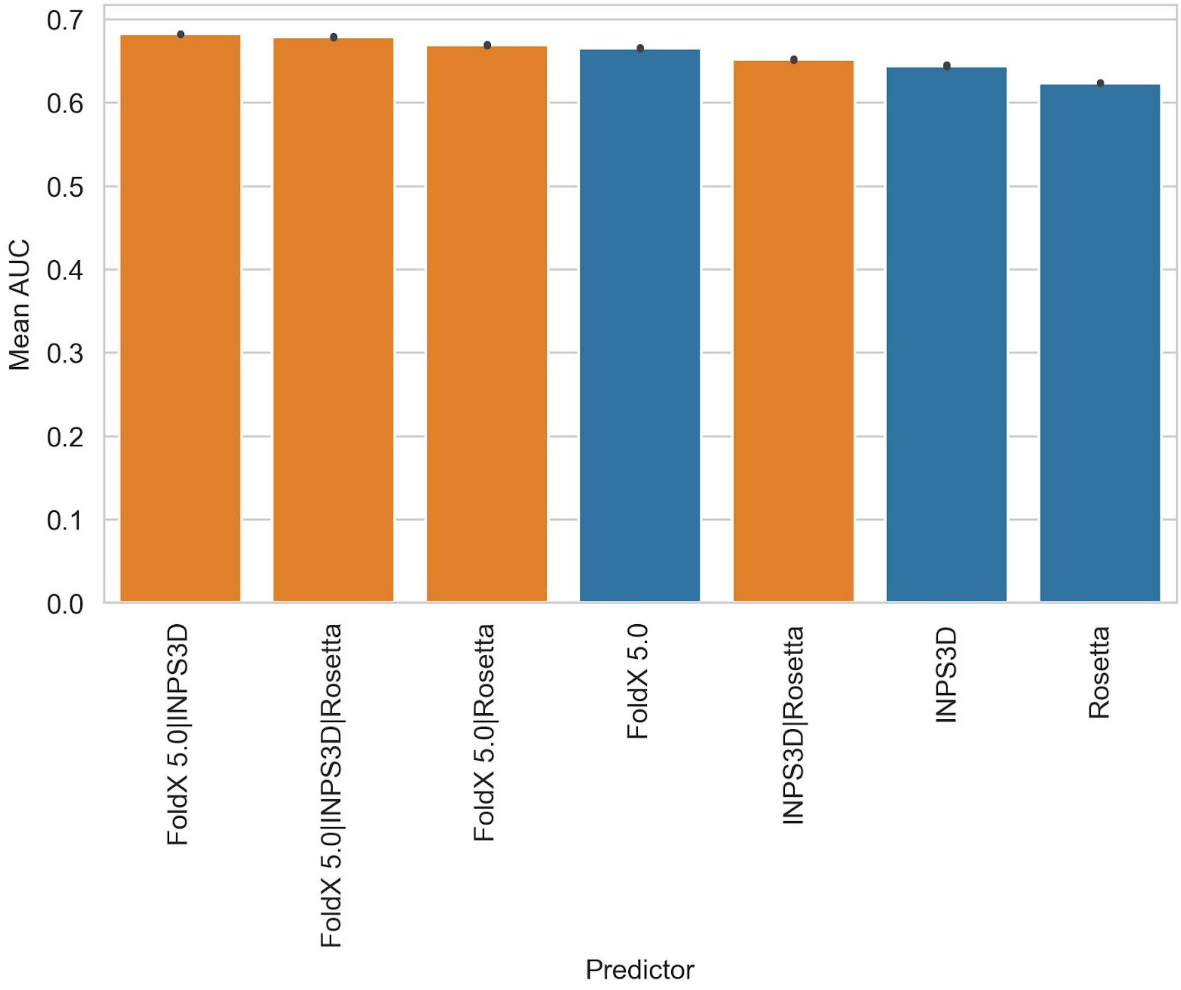

Figure S1: Barplots displaying the performance (AUC) of the best three performing ΔΔG predictors (blue) and their consensuses (orange). The bars represent the mean AUC obtained by averaging balanced subsets (the available pathogenic variants were matched with a random sample with the same number of benign variants for one hundred times).

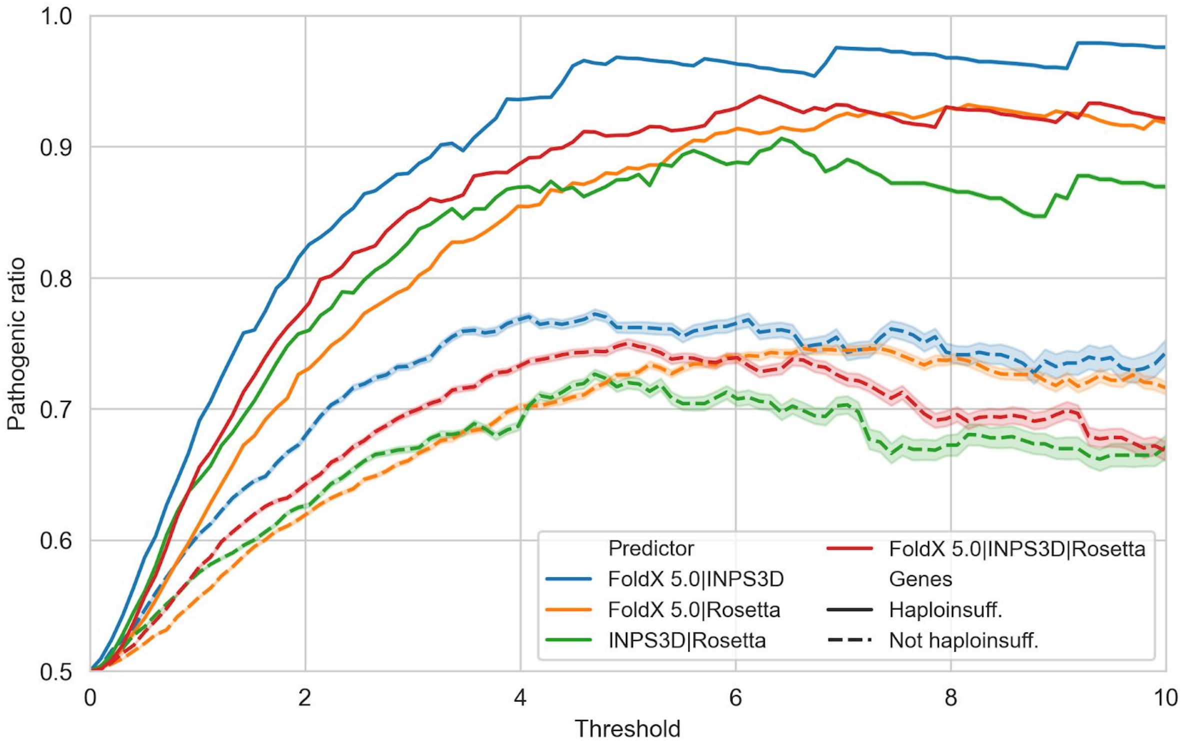

Figure S2: Precision (y-axis) of the consensuses of the best three performing stability-based methods in predicting pathogenicity at different |ΔΔG| values, defined as the ratio of truly pathogenic over all the variants reporting predicted |ΔΔG| values above a specific threshold (x-axis). Solid and dashed lines are computed on variants in haploinsufficient and non-haploinsufficient genes, respectively. The best performing method consists of the FoldX and INPS3D average.

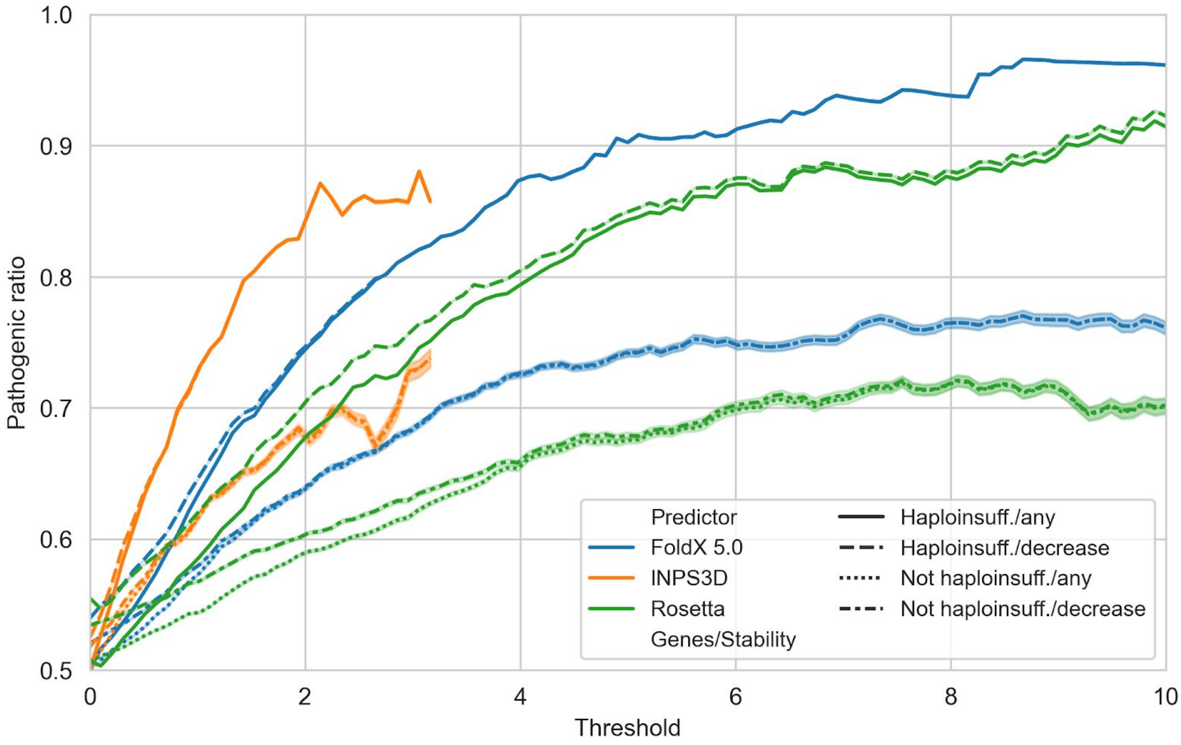

Figure S3: Precision (y-axis) of the best three performing stability-based methods in predicting pathogenicity at different |ΔΔG| values, defined as the ratio of truly pathogenic over all the variants reporting predicted |ΔΔG| values above a specific threshold (x-axis). Differently dashed lines show the precision for variants in haploinsufficient and non-haploinsufficient genes and that cause stability changes in any direction or only stability decrements (destabilizing variants).

Discarding variants that increase stability (stabilizing variants) slightly increase the enrichment of pathogenic variants for all predictors in both haploinsufficient and non-haploinsufficient genes. However, the differences are only appreciable for lower |ΔΔG| thresholds.
